# On the Treatment of Febrile Diseases

**Published:** 1799-11

**Authors:** Edward Miller

**Affiliations:** New York


### *On the Treatment of Febrile Diseases, by Dr.* EDWARD MILLER, *of New York.*

[Extracted from a Paper of the same Author, "*on Cutaneous Perspiration, and the Operation and Uses of Specific Remedies.*" Comp. our Journ. Vol. I. No. III. p. 287.]

AFTER attempting to restrict the use of sudorific remedies to such narrow limits, it may not be improper to recall the reader's attention to a substitute better adapted to the nature, circumstances, and varieties of fevers. This substitute is water, of various temperature, taken into the stomach, injected into the bowels, and applied to the surface of the body. Many endeavours have been made to bring this inestimable remedy into more common use; hitherto, indeed, without much success; but it is to be hoped that the time now approaches, when its efficacy will no longer be disdained on account of its simplicity and cheapness.

The causes of fever would be infinitely less pernicious to the system, if the fever itself were repressed in its first movement, or annihilated in embryo. The cool treatment of the small pox, gives an example of this suppression of a disease; but physicians have never yet sufficiently availed themselves of the instruction it affords. Notwithstanding all the complicated maxims and rules of medical practice, the genuine treatment of fevers is simple; it chiefly consists in reducing the heat of the system, when too high, and increasing it when too low: the former will allay the excessive action, which threatens organic destruction of the more important and delicate viscera, or an eventual exhaustion of the principle of life; and the latter will obviate such accumulation of excitability, as may endanger the system from the violence of subsequent re-action.

[ To be concluded in our next Number. ]

